# An Improved Methodology to Overcome Key Issues in Human Fecal Metagenomic DNA Extraction

**DOI:** 10.1016/j.gpb.2016.06.002

**Published:** 2016-11-23

**Authors:** Jitendra Kumar, Manoj Kumar, Shashank Gupta, Vasim Ahmed, Manu Bhambi, Rajesh Pandey, Nar Singh Chauhan

**Affiliations:** 1 Department of Biochemistry, Maharshi Dayanand University, Rohtak 124001, India; 2 Ayurgenomics Unit, Translational Research and Innovative Science ThRough Ayurgeomics, Council of Scientific and Industrial Research, Institute of Genomics and Integrative Biology, New Delhi 110020, India

**Keywords:** Metagenomic DNA extraction, Gut microbiome, Human feces, *16S* rRNA

## Abstract

Microbes are ubiquitously distributed in nature, and recent culture-independent studies have highlighted the significance of gut microbiota in human health and disease. Fecal DNA is the primary source for the majority of human **gut microbiome** studies. However, further improvement is needed to obtain fecal metagenomic DNA with sufficient amount and good quality but low host genomic DNA contamination. In the current study, we demonstrate a quick, robust, unbiased, and cost-effective method for the isolation of high molecular weight (>23 kb) metagenomic DNA (260/280 ratio >1.8) with a good yield (55.8 ± 3.8 ng/mg of feces). We also confirm that there is very low human genomic DNA contamination (eubacterial: human genomic DNA marker genes = 2^27.9^:1) in the **human feces**. The newly-developed method robustly performs for fresh as well as stored fecal samples as demonstrated by ***16S* rRNA** gene sequencing using 454 FLX+. Moreover, *16S* rRNA gene analysis indicated that compared to other DNA extraction methods tested, the fecal metagenomic DNA isolated with current methodology retains species richness and does not show microbial diversity biases, which is further confirmed by qPCR with a known quantity of spike-in genomes. Overall, our data highlight a protocol with a balance between quality, amount, user-friendliness, and cost effectiveness for its suitability toward usage for culture-independent analysis of the human gut microbiome, which provides a robust solution to overcome key issues associated with fecal metagenomic DNA isolation in human gut microbiome studies.

## Introduction

Humans live in close association with microbes which act as a constituent organ [1], [2]. The total number of microbes residing in the human body, especially in the gut, outnumbers that of human cells [3]. A vast array of recent studies has identified many microbial enterotypes in the human gut [4], [5], [6], [7], [8], [9], [10] and their potential roles in immunity [5], [7], [8], development [8], digestion [9], and other functions [10].

Majority of the studies have used *16S* rRNA gene sequencing to understand the community structure, composition, and functional diversity of the human gut microbiome [1], [2], [3], [4]. The success of these culture-independent studies depends primarily on the quality and quantity of metagenomic DNA isolated from the given samples [11], [12], [13]. Therefore, isolation of metagenomic DNA with a good quality from a heterogeneous source like human feces has been a challenging task.

Human feces are complex due to the presence of fibers, microbes, undigested particles, nucleases, and human cells [14]. Removal of fibers and undigested particles from feces is difficult, which in turn affects overall quality and quantity of metagenomic DNA being isolated [14], [15], [16]. Moreover, the presence of these impurities also compromises efficient lysis of microbial cells. Many microbes elude complete lysis, resulting in an uneven contribution of metagenomic DNA and eventually compromised microbial diversity [11], [17]. Additionally, human genomic DNA remnants in metagenomic DNA affect the metagenomic sequence data output, thereby increasing per base sequencing cost [6]. To overcome these challenges, many metagenomic DNA extraction protocols are being standardized, which include phenol/chloroform enzymatic lysis and freeze thaw [11], [16], [17], [18], [19], [20], [21], [22]. These kits have improved the quality of metagenomic DNA. However, great concerns remain pertaining to microbial diversity biasness and human genomic DNA contamination [12], [17], [20]. The cost per sample, amount of sample, and associated impurities are other issues which may be improved upon. In the current study, we intend to overcome these limitations and provide a faster, robust, and economical metagenomic DNA extraction method with a good quality and quantity.

## Methods

Fresh human fecal samples were collected from healthy individuals into a sterile container and stored at −86 °C until use. Human ethical guidelines were followed strictly before engaging individuals for sample collection. The study has been conducted after ethical clearance from human ethics committee of Maharshi Dayanand University, Rohtak, Haryana, India.

### Isolation of metagenomic DNA from fecal samples with current method

The current methodology comprises two steps: (1) purification of the microbial cells from fecal impurities and (2) lysis of microbial cells to obtain metagenomic DNA with high molecular weight.

At the first step, fresh feces (100 mg) were weighed into a sterile microcentrifuge tube for isolation of purified microbial cells. Microbial cells were sequentially washed with normal saline solution (0.9% NaCl solution) and phosphate-buffered saline (PBS; pH 7.4). The washing steps were optimized for the recovery of a purified bacterial pellet to obtain quality fecal metagenomic DNA for the downstream studies. In 5 sets of replicates, 100 mg of feces were resuspended in 1 ml of normal saline solution by vortexing for 30 s and then centrifuged at ambient room temperature (RT) for 2 min with different speed of 1000 rpm (72 × *g*), 2000 rpm (287 × *g*), 3000 rpm (645 × *g*), 4000 rpm (1147 × *g*), and 5000 rpm (1792 × *g*), respectively. The resulting supernatants were subjected to microscopic examination for the presence of fibers and insoluble impurities. Recovered supernatant was centrifuged again at 10,000 rpm (7168 × *g*) for 1 min at ambient room temperature to collect microbial pellet for downstream processing. Microbial pellet from all replicates was subsequently washed with 1 ml of PBS (pH 7.4) for centrifugation with different speeds as described above. The resulting supernatants were subjected to centrifugation again at 10,000 rpm (7168 × *g*) for 1 min to recover microbial pellet, which was used for metagenomic DNA isolation at the next step.

At the second step, the purified microbial pellet was resuspended in 500 μl of lysis buffer containing 1% (w/v) cetyl trimethyl ammonium bromide (CTAB), 100 mM of ethylenediaminetetraacetic acid (EDTA), 1.5 M of NaCl, 100 mM of Na_3_PO_4_, and 100 mM of Tris–HCl (pH 8.0). After adding 2 μl of proteinase K (20 mg/ml), the mixture was incubated for 10 min at 37 °C with gentle shaking at 100 rpm in orbital shaker incubator. Afterward, sodium dodecyl sulfate (SDS) was added with a final concentration of 1% and the incubation continued for another 20 min at 65 °C with intermittent shaking. The lysate was centrifuged at 13,000 rpm (12,114 × *g*) for 5 min at ambient room temperature. The resulting supernatant was collected and mixed with an equal volume of saturated phenol:chloroform:isoamyl alcohol (25:24:1), which is then subjected to centrifugation at 10,000 rpm (7168 × *g*) for 5 min at RT. The aqueous phase was collected and metagenomic DNA was precipitated with 0.6 volume of isopropanol and pelleted by centrifugation at 13,000 rpm (12,114 × *g*) for 5 min. After washing twice with 70% ethanol, the resulting DNA was dried and finally dissolved into a 50 μl of 1 × Tris–EDTA buffer (pH 8.0).

**Figure 1:**
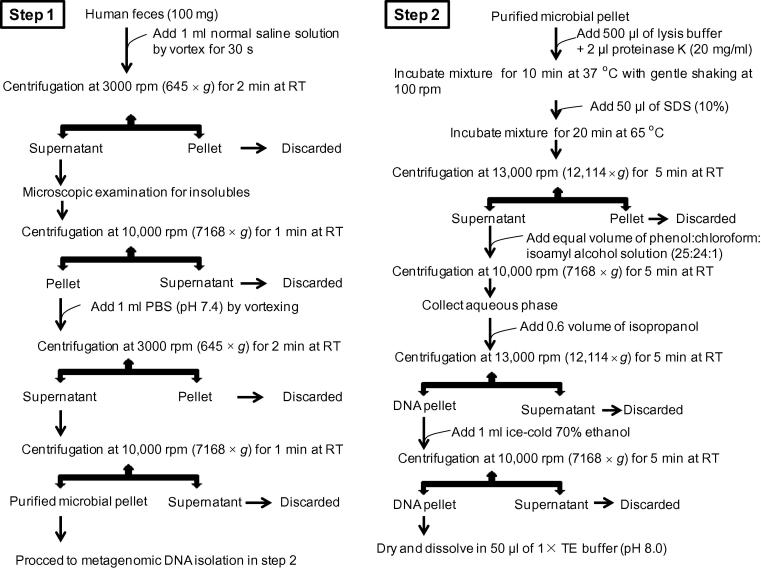
**Workflow for fecal metagenomic DNA extraction using the current method**

The qualitative and quantitative analysis of the metagenomic DNA was performed by agarose gel electrophoresis, restriction endonuclease digestion (*Sau*3A1), NanoQuant (Tecan Group, Mannedorf, Switzerland) estimation, and Qubit® dsDNA HS Assay Kit (Life Technologies, Carlsbad, CA). Metagenomic DNA recovered from all replicates was compared for qualitative and quantitative parameters to obtain optimized condition for metagenomic DNA isolation from human feces. The optimized method is outlined in Figure 1 and was then used to isolate the metagenomic DNA from 10 one-month-old frozen feces stored at −86 °C and 50 random fecal samples (including both fresh and frozen samples) to evaluate its robustness.

### Isolation of metagenomic DNA from fecal samples with commercial methods/kits

Metagenomic DNA was isolated from fresh or frozen human fecal samples using 4 commercial kits, including Power Fecal® DNA Isolation Kit (MO BIO Laboratories, Carlsbad, CA) [11] (referred as method A hereafter), Extract Master™ Fecal DNA Extraction Kit (Epicentre, Madison, WI) [21] (referred as method B hereafter), Favor Prep™ Stool DNA isolation Kit (Favorgen Biotech, Ping-Tung, Taiwan, China) (referred as method C hereafter), and QIAamp DNA Stool Kit (QIAGE, Hilden, Germany) (referred as method D hereafter) as instructed by the respective manufacturers. The quality and quantity of fecal metagenomic DNA were assessed as mentioned above.

### qPCR amplification

The qPCRs were performed on a 7500 Fast Real Time PCR system (ABI, Life Technologies, Carlsbad, CA) using 2 × KAPA SYBR Fast qPCR master mix (universal) from KAPA Biosystems (Wilmington, MA). The 20 μl reaction mixture contained 1 μl of metagenomic DNA/human genomic DNA (30 ng/μl), 7.5 μl of 2 × SYBR Green, 1 μl of primer mix (forward and reverse primer of 0.5 mM), 0.4 μl of master mix (High Rox), and 5.1 μl of nuclease-free water. Primers used include human *MUC5B*-specific primers and eubacterial *16S* rRNA gene-specific primers (Table S1). The experiment was performed in triplicate using human genomic DNA as control. The qPCRs were performed with holding stage at 95 °C for 20 s, 40 cycles of 95 °C for 30 s, and 60 °C and final melt curve stage with continuous mode at 95 °C for 15 s, 60 °C for 60 s, 95 °C for 15 s, and 60 °C for 15 s. Melt curve analysis of the primers confirmed the high efficiency of the primers and amplified product generated during the reaction. The relative quantification was carried out using the 2^−ΔΔCT^ method.

Efficacy of current methodology was further validated with fecal samples spiked with *Escherichia coli* DH10B (Invitrogen, Carlsbad, CA), *Bacillus subtilis* (MTCC-2057, Chandigarh, India), and *Aspergillus niger* (MTCC-514, Chandigarh, India). The efficacy of current methodology was analyzed by quantitatively comparing the presence of marker genes of *16S* rRNA and internal transcribed spacer (ITS) in DNA extracted using current methodology from spiked stool samples and DNA extracted from respective pure cultures. All DNA quantification experiments were performed with host-specific primers including *16S* rRNA gene primers (16S120_FP and 16S345_RP) for microbes and *ITS* gene primers (ITS 1F and ITS 4B) for fungus [23] using qPCR with aforementioned PCR settings.

### Pyrosequencing and sequence analysis of *16S* rRNA gene

The *16S* rRNA gene was amplified from metagenomic DNAs extracted with different methodologies following optimized PCR conditions [24]. The resulting amplicons were analyzed using agarose gel electrophoresis and quantified with Qubit® dsDNA HS Assay Kit.

The amplified *16S* rRNA gene from the metagenomic DNA isolated with our methodology and with method A were also sequenced with Roche 454 GS FLX+ system, following the manufacturer’s recommendations. The *16S* rRNA gene sequences generated and used in the current study were submitted as a NCBI Bioproject (Accession ID: PRJNA295000). Subsequently, Quantitative Insights Into Microbial Ecology (QIIME) pipeline was implemented for pyrosequencing data analysis [24], along with *16S* rRNA gene sequence data obtained from the Human Microbiome Project (HMP) [25]. Variability analysis of *16S* rRNA gene sequences was performed using QIIME statistical tools [24], [25], [26].

## Results and discussion

### Isolation of metagenomic DNA from human feces

A number of efforts to optimize a methodology for metagenomic DNA isolation from feces have been undertaken [11], [13], [15], [20]. Although progress has been made in this regard, problems of limited applicability (*e.g.*, microbial diversity studies only) and acceptability due to their complex process, poor metagenomic DNA quality, host genomic DNA contamination, low yield, and high cost have still left scope for a methodology to overcome the shortcomings [11], [17], [18], [19], [20], [21].

The current methodology is a two-step process with hands-on time of 80–90 min. At the first step, various large size insoluble impurities like undigested food particles and dietary fibers were removed to collect a clean translucent microbial pellet. Purification of microbial pellet would enable efficient lysis of microbial cells and a better DNA recovery. In the second step, the microbial cells were treated with lysis buffer and proteinase for microbial cell lysis to achieve a high yield of metagenomic DNA. The microscopic examination showed that feces washing and following centrifugation at 3000 rpm (645 × *g*) removed majority of insoluble impurities with a minimum microbial loss. While washing and following centrifugation at 1000 rpm (72 × *g*) and 2000 rpm (287 × *g*) enabled maximum microbial recovery with abundant insoluble impurities, washing and following centrifugation at 4000 rpm (1147 × *g*) and 5000 rpm (1792 × *g*) have removed all impurities with huge microbial loss. Among all replicates, feces washing and following centrifugation at 3000 rpm (645 × *g*) yielded good quality of purified microbial pellet, and subsequently metagenomic DNA with high molecular weight free from molecular inhibitors of comparable yield (Table S2).

**Figure 2:**
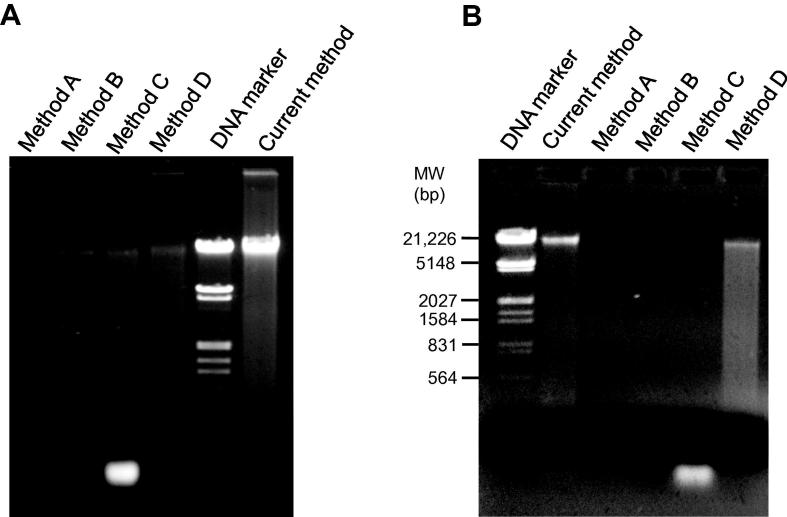
**Gel electrophoresis of human fecal metagenomic DNA isolated with different methods** Metagenomic DNA isolated from fresh human feces (**A**) or one-month-old human feces stored at −86 °C (**B**) using different methods was separated on 0.8% agarose gel. Phage *λ* DNA *Eco*R1/*Hin*dIII digest was used as DNA marker. Method A, Power Fecal® DNA Isolation Kit from MO BIO Laboratories; method B, Extract Master™ Fecal DNA Extraction Kit from Epicentre; method C, Favor Prep™ Stool DNA isolation Kit from Favorgen Biotech; method D, QIAamp DNA Stool Kit from QIAGEN; current method, method presented in the current study. Metagenomic DNA was extracted and eluted per manufacturers’ instruction. The volume of DNA elutions was normalized according to the starting amount of fecal samples and then equal volume of DNA was used for uniform sample loading to perform comparison among different methods.

The DNA yield was 55.80 ± 3.80 ng/mg of feces (Table 1). Spectrometric analysis using NanoQuant showed 260/280 ratio of 1.83 ± 0.02. Qualitative analysis with agarose gel electrophoresis also confirmed good integrity for DNA with size >23 kb (Figure S1) and negligible RNA presence (Figure2**A**).

The robustness of the protocol was also confirmed using one-month-old frozen fecal sample stored at −86 °C with a yield of 40.00 ± 5.00 ng/mg of feces. The protocol was repeated for an independent set of 50 non-redundant fecal samples with varying texture and consistency. The quality (260/280 ratio of 1.8–1.9) and quantity (47.5 ± 2.5 ng/mg of feces) was consistent within all replicates (Table 1).

In summary, current methodology has been successfully used to isolate the metagenomic DNA from one-month-old frozen fecal sample stored at −86 °C with a minimal effect on yield and quality, which was a challenge as per reported metagenomic DNA isolation studies [13], [14], [20], [21], [22].

### Comparative analysis with commercial methods/kits

To evaluate the relative performance of our current method, we compared it with other commercially available metagenomic DNA isolation kits for human feces [11], [16], [21]. As instructed by kit manufacturers, various defined amount of fecal sample were used for commercial methods A, B, C, and D. Notably, low yields of DNA were obtained using these methods in comparison to that using current method (Table 1). Gel electrophoresis analysis indicated poor DNA yield with method A, B, and D, while RNA contamination was observed in DNA isolated with method C (Figure2A). Similarly, spectrometric analysis showed a lower yield and compromised 260/280 ratios for DNA extracted using methods A−D (Table 1). Moreover, these methods did not work well for extracting metagenomic DNA from one-month-old stored fecal sample (Table 1). We failed to extract metagenomic DNA from frozen feces using methods A–C, while a low amount of DNA was recovered using method D. On the other hand, although with a reduced yield when compared to using fresh feces, more significant DNA with decent 260/280 ratio was recovered when using our method to extract DNA from frozen feces. In contrast, a poor quality of metagenomic DNA with negligible output was observed with one-month-old frozen feces at −86 °C with all other methods tested (Figure2**B**).

The metagenomic DNA isolated from fresh human fecal samples with current method and other commercial methods was further analyzed for contamination of host genomic DNA using qPCR. The amplification of eubacterial and human genomic DNA marker genes, *16S* and *MUC5B*, indicated a huge difference in the copy number of eubacterial:human genes (2^27.9^:1) in the metagenomic DNA isolated with current method. However, the eubacterial:human ratios were compromised for the fecal metagenomic DNA isolated using commercial methods (2^13^:1 for method A, 2^12^:1 for method B, and 2^0.143^:1 for method C, 2^20.8^:1 for method D). Similar observation on host genomic DNA contamination in fecal metagenomic DNA was recorded during the HMP study using method A [6].

The cycle threshold (Ct) value is used for absolute copy number quantification of a target gene in qPCR. In general, lower Ct value indicates higher copy number of target gene, while higher Ct value means low copy number of target gene. A low Ct value of 6.780 ± 0.231 was observed for eubacterial *16S*, and of 34.740 ± 0.374 for human-specific *MUC5B* using metagenomic DNA isolated with current method. Metagenomic DNA isolated with other methods showed varied Ct values: 19.310 ± 0.185 (method A), 22.010 ± 0.089 (method B), 28.130 ± 0.821 (method C), and 11.780 ± 0.295 (method D) for *16S* gene, respectively, while Ct values of 32.690 ± 0.332 (method A), 34.090 ± 0.166 (method B), 28.270 ± 3.426 (method C), and 32.630 ± 0.647 (method D) were observed for human-specific *MUC5B* gene, respectively. Lower Ct values of other methods for human-specific *MUC5B* gene compared to our method indicated few human DNA remnants in the metagenomic DNA isolated with current protocol, reflecting superior representation of the eubacterial-specific metagenomic DNA in comparison to the limited presence of human genomic DNA in metagenomic DNA extracted with current methodology.

qPCR analysis of known genome spike-in experiments was further performed for validation of the efficacy and unbiased cellular lysis of current methodology. qPCR analysis indicated a good recovery of 79.85 ± 12.16% for *E. coli* DH10B genomic DNA in fecal samples spiked with *E. coli* DH10B and 72.09 ± 5.02% for *B. subtilis* genomic DNA in fecal samples spiked with *B. subtilis*. However, a slightly lower recovery (36.35 ± 9.49%) was observed for *A. niger* genomic DNA in fecal samples spiked with *A. niger*. A good amplification observed with the metagenomic DNA isolated using current method indicates the fecal metagenomic DNA isolated with current methodology was free from the impurities that could interfere the reaction [17], [19], [21].

### Evaluation of current method for human microbiome studies by pyrosequencing

To further evaluate the applicability of the isolated DNA samples for sequencing analysis, the *16S* rRNA gene (V1–V4 region) was amplified from metagenomic DNA isolated using our protocol and method A after column purification. These amplicons were subjected to sequencing analysis using Roche 454 FLX+. As a result, we obtained 57,689 sequencing reads with an average read length of ∼530 bp for the 4 samples tested. These sequences were quality filtered (>Q30) to remove ambiguous and chimeric sequences. Finally, 54,262 high-quality reads were retained for the following downstream analysis.

As shown in Table 2, more *16S* rRNA gene sequencing reads were recovered for DNA isolated using method A than using current method. The reads were processed using QIIME *de novo* clustering pipeline to get the operational taxonomic units (OTUs). Similarly, we found that more OTUs were detected for DNA isolated using method A than using current method (Table 2). We then estimated the species richness by normalizing the read counts with the sequence value (−e) based on the minimum number of high quality sequencing reads for each sample, which is 6300 in the current study, and analyzed the total alpha diversity. Our results showed that despite fewer reads, more species were observed for DNA isolated using current method than using method A. An average number of 611 and 635 microbial species were observed in samples H1 and H2, respectively, which were isolated using the current method, in comparison to 515 and 496 microbial species from the same source feces with DNA isolated using method A (Table 2). In the meantime, we also noticed a higher Shannon diversity index. Taken together, these data indicate that even for the same fecal samples, sequencing outcomes can be greatly affected by the metagenomic DNA isolation methods used [12].

**Figure 3:**
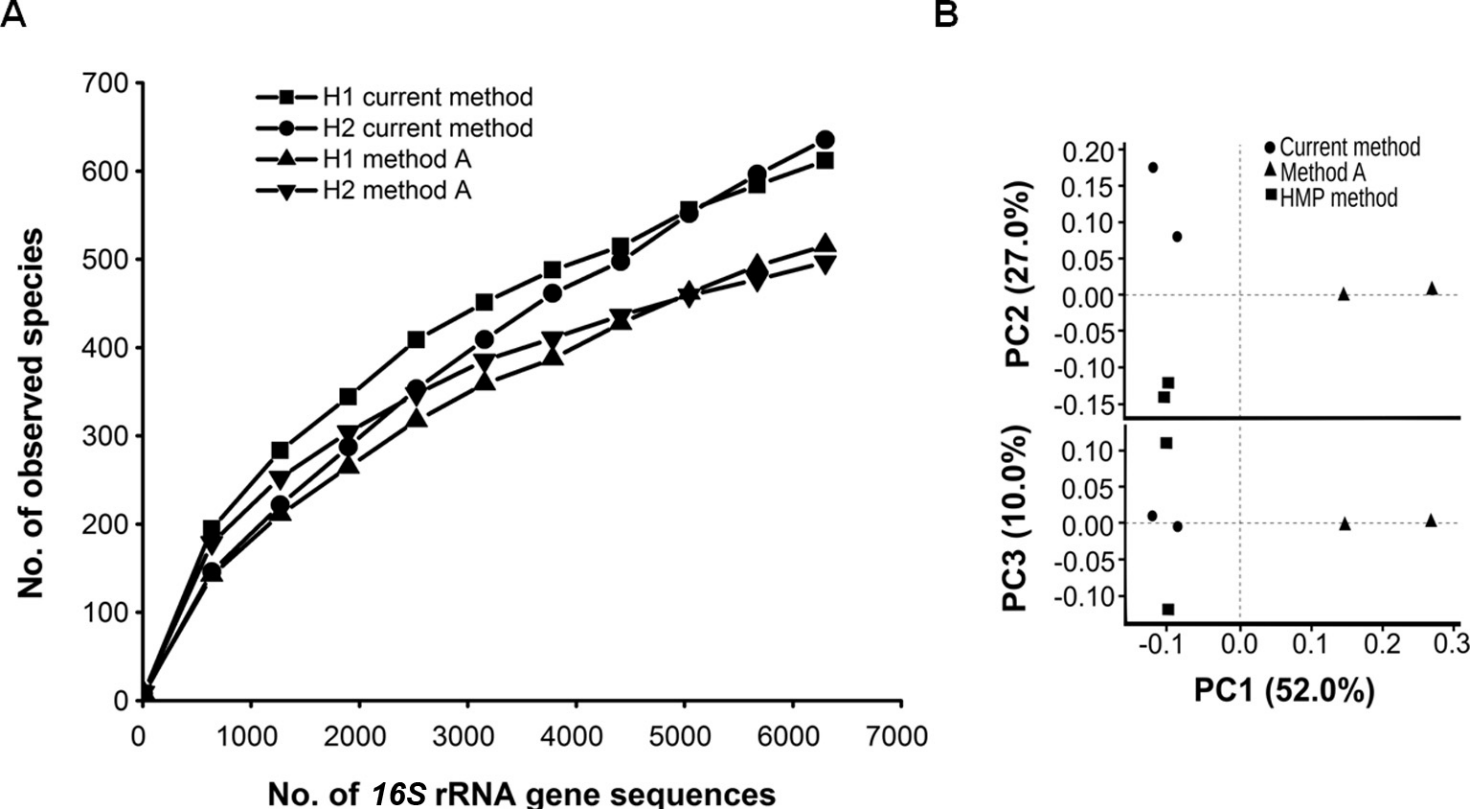
**Diversity analyses of *16S* rRNA gene sequencing readsA.** α diversity analysis based on *16S* rRNA gene sequencing reads amplified from metagenomic DNA isolated from samples H1 and H2 using current method and method A. **B.** Principal coordinate analysis plots show the β diversity analysis based on *16S* rRNA gene sequencing reads amplified from metagenomic DNA isolated from samples H1 and H2 using current method (●), method A (▲), and the HMP sequencing data (SRS019543 and SRS018968) (■). Pyrosequencing was performed on Roche 454 FLX+ platform for PCR amplicons using fecal metagenomic DNA as template. H1 and H2 represent fresh human feces of two healthy individuals. HMP, Human Microbiome Project.

Number of observed species relative to the increasing number of *16S* rRNA gene sequencing reads obtained was also analyzed to obtain the identification rate of new OTUs (Figure3**A**). The identification rate of microbial phylotypes was slightly higher for *16S* sequencing reads generated from metagenomic DNA extracted with current method than method A. These results highlight the usefulness of the current method to capture species richness even from lower number of sequencing reads. More observed species with better identification rate can be obtained from metagenomic DNA isolated using current methodology (Figure3A).

Biasness has been reported between microbial diversity of a host sample and its metagenomic DNA extraction method [12], [22]. To test whether there exist such biases in current methodology, a β diversity analysis was performed between the microbial diversities obtained from DNA isolated with current method, HMP data, and method A. The β diversities show positioning of the samples based on their microbial diversities. Principal coordinate analysis (PCoA) plots were generated from *16S* rRNA gene sequencing reads (Figure3**B**). Compared to method A, significant variability of inter-individual microbial diversity was observed for the current method. It indicates that the current methodology performed better with respect to unbiased lysis of microbial cells and contribution into the total metagenomic DNA pool.

Overall, the results we presented here highlight the efficiency of the current protocol to achieve better yield and quality of the gut metagenomic DNA while simultaneously retaining the sample enrichment with respect to the bacterial species present in the human gut. The current protocol has not been tested for other host species. Different host species are of specific diet pattern and life style, which in turn affect the constituents of the feces. Therefore, the protocol presented here may warrant specific but minor adjustments to account for species-specific gut metagenomic DNA isolation. For most of the species, the requirement may be met by maneuvering the relative concentration of NaCl and the centrifugation conditions which are important for purifying microbial cells from the background impurities. Given the user-friendliness of the current protocol, this may be optimized at individual level without great difficulties.

## Authors’ contributions

JK, MK, and VA performed sample collection, DNA isolation, and experiment optimization. RP performed real-time PCR analysis and *16S* rRNA gene sequencing. SG carried out sequence analysis. MB and NSC designed the study and drafted the manuscript. All authors read and approved the final manuscript.

## Competing interests

The authors have declared that there are no competing interests.

## Figures and Tables

**Table 1:** Qualitative and quantitative analysis of human fecal metagenomic DNA extraction using different methods

**Fecal sample**	**Extraction method**	**Amount of sample tested (mg)**	**Total extraction time (min)**	**260/280 ratio**	**DNA yield (ng/mg of feces)**
Fresh	Current method	100	80–90	1.83 ± 0.02	55.80 ± 3.80
	Method A	250	120	1.28 ± 0.12	3.40 ± 0.60
	Method B	50	120	1.79 ± 0.04	1.10 ± 0.20
	Method C	100	90–150	1.81 ± 0.64	15.50 ± 0.50
	Method D	200	60–90	1.77 ± 0.28	6.79 ± 0.43
Frozen	Current method	100	80–90	1.89 ± 0.06	40.00 ± 5.00
	Method A	250	120	–	–
	Method B	50	120	–	–
	Method C	100	90–150	–	–
	Method D	200	60–90	1.48 ± 0.07	1.60 ± 0.60

*Note:* The metagenomic DNA was isolated in triplicate with all methodologies per manufacturers’ instruction and dissolved in the Tris–EDTA buffer (pH 8.0).

**Table 2:** Microbial diversity analysis of the *16S* rRNA gene sequencing reads

**Sample**	**No. of reads**	**Average read length (bp)**	**No. of detected OTUs**	**Average No. of observed species**	**Average Shannon diversity index**
H1 current method	8602	530	696	∼611	6.346
H2 current method	6323	530	497	∼635	6.852
H1 method A	16,640	530	870	∼515	5.795
H2 method A	22,697	530	844	∼496	5.245

*Note:* H1 and H2 represent metagenomic DNA isolated from fresh human feces of healthy individuals. OTU, operational taxonomic unit.

## Supplementary material

Supplementary Figure S1 Gel electrophoresis of fresh and frozen human fecal metagenomic DNA Metagenomic DNA isolated from fresh human feces (lane 1) or one-month-old human feces stored at –86 °C (lane 2) using current method was separated on 0.8% agarose gel. NEX-GEN DNA ladder (EXL)(Puregene, Genetix Biotech, India) was used as DNA marker.

Supplementary Table S1 List of primers used in current study

Supplementary Table S3 Comparative analysis of the metagenomic DNA isolated under different microbial purification conditions
