# Supplementary figures and images for: An Improved Methodology to Overcome Key Issues in Human Fecal Metagenomic DNA Extraction
Source: Genomics Proteomics Bioinformatics. 2016 Nov 23;14(6):371–8. doi: 10.1016/j.gpb.2016.06.002 (PMC5200916; doi:10.1016/j.gpb.2016.06.002)

## Slide 1
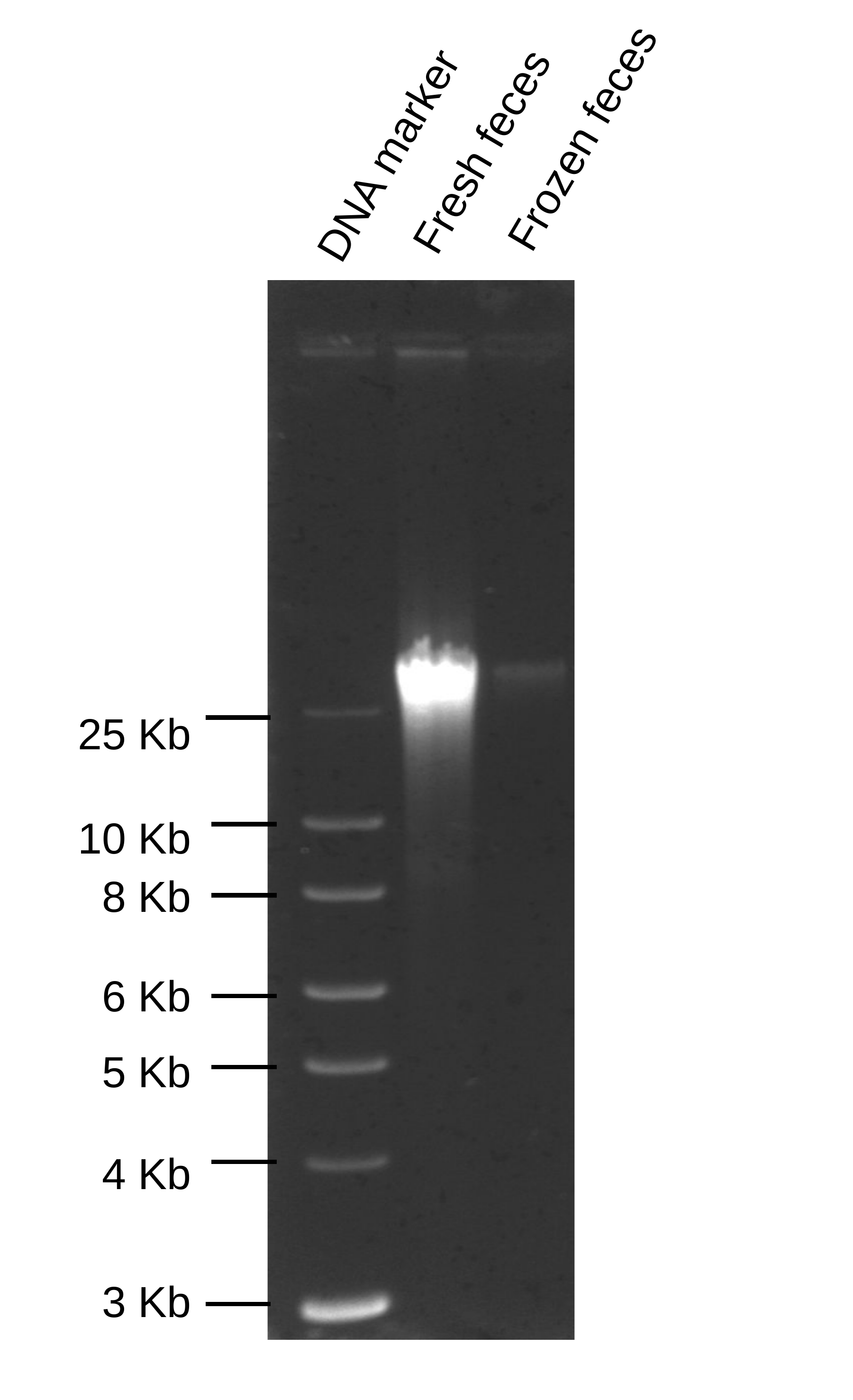

DNA marker
Frozen feces
Fresh feces
25 Kb
10 Kb
8 Kb
6 Kb
5 Kb
4 Kb
3 Kb

Supplement: Supplementary Figure S1 — Gel electrophoresis of fresh and frozen human fecal metagenomic DNA Metagenomic DNA isolated from fresh human feces (lane 1) or one-month-old human feces stored at –86 °C (lane 2) using current method was separated on 0.8% agarose gel. NEX-GEN DNA ladder (EXL)(Puregene, Genetix Biotech, India) was used as DNA marker. [file mmc1.pptx]
