# Supplementary material for: An Improved Methodology to Overcome Key Issues in Human Fecal Metagenomic DNA Extraction
Source: Genomics Proteomics Bioinformatics. 2016 Nov 23;14(6):371–8. doi: 10.1016/j.gpb.2016.06.002 (PMC5200916; doi:10.1016/j.gpb.2016.06.002)
Supplement: Supplementary Table S1 — List of primers used in current study [file mmc2.docx]

**Table S1 List of primers used in current study**

| **Primer name** | **Target gene** | **Primer sequence (5′–3′)** |
| --- | --- | --- |
| MUC5B-FP | Human *MUC5B* | ACGTCAAGGCCACAGCTATT |
| MUC5B-RP | Human *MUC5B* | AGGTGGGAGGCTCCTCTG |
| 16S120_FP | Eubacterial *16S* rDNA | ACTGGCGGACGGGTGAGTAA |
| 16S345_RP | Eubacterial *16S* rDNA | TCCTTACTGCTGCCTCCCG |
| ITS 1F | *ITS* | CTTGGTCATTTAGAGGAAGTAA |
| ITS 4B | *ITS* | CAGGAGACTTGTACACGGTCCAG |

*Note*: MUC5B, mucin 5B; ITS, internal transcribed spacer.
