# Supplementary material for: An Improved Methodology to Overcome Key Issues in Human Fecal Metagenomic DNA Extraction
Source: Genomics Proteomics Bioinformatics. 2016 Nov 23;14(6):371–8. doi: 10.1016/j.gpb.2016.06.002 (PMC5200916; doi:10.1016/j.gpb.2016.06.002)
Supplement: Supplementary Table S3 — Comparative analysis of the metagenomic DNA isolated under different microbial purification conditions [file mmc3.docx]

**Table S2 Comparative analysis of the metagenomic DNA isolated under different microbial purification conditions**

| Centrifugation | 260/280 ratio | DNA concentration (ng/µl) | Relative *Sau*3A digestion (%) | PCR amplification | Cells recovered after washing (%) | Relative abundance of impurities in supernatant |
| --- | --- | --- | --- | --- | --- | --- |
| 1000 rpm (72 × *g*) | > 1.7 | 98.6 ± 0.4 | 76 | Poor | ~86 | +++ |
| 2000 rpm (287 × *g*) | > 1.8 | 70.5 ± 0.5 | 84 | Good | ~70 | ++ |
| 3000 rpm (645 × *g*) | > 1.9 | 51.5 ± 0.5 | 100 | Very good | ~60 | + |
| 4000 rpm (1147 × *g*) | > 1.9 | 33.0 ± 1.0 | 100 | Very good | ~40 | + |
| 5000 rpm (1792 × *g*) | > 1.9 | 38.4 ± 0.8 | 100 | Very good | ~20 | - |

*Note*: Data were obtained during optimization of metagenomic DNA isolation from fresh and frozen human feces.
